# Biotin Deficiency Induces Intestinal Dysbiosis Associated with an Inflammatory Bowel Disease-like Phenotype

**DOI:** 10.3390/nu15020264

**Published:** 2023-01-04

**Authors:** Julianne C. Yang, Jonathan P. Jacobs, Michael Hwang, Subrata Sabui, Fengting Liang, Hamid M. Said, Jonathan Skupsky

**Affiliations:** 1 The Vatche and Tamar Manoukian Division of Digestive Diseases, Department of Medicine, David Geffen School of Medicine, University of California, Los Angeles, CA 90095, USA; 2 Division of Gastroenterology, Department of Medicine, VA Greater Los Angeles Healthcare System, Los Angeles, CA 90073, USA; 3 Department of Physiology and Biophysics, University of California, Irvine, CA 92697, USA; 4 Department of Medicine, University of California, Irvine, CA 92697, USA; 5 Division of Gastroenterology, Department of Medicine, Tibor Rubin VA Medical Center, Long Beach, CA 90822, USA

**Keywords:** microbiome, biotin, IBD, colitis

## Abstract

Biotin is an essential vitamin and critical cofactor in several metabolic pathways, and its deficiency has been linked to several disorders including inflammatory bowel disease (IBD). We previously reported that biotin deficiency (BD) in mice, whether modeled through intestine-specific deletion of biotin transporter (SMVT-icKO) or through a biotin-deficient diet, resulted in intestinal inflammation consistent with an IBD-like phenotype. To assess whether the gut microbiome is associated with these BD-induced changes, we collected stool and intestinal samples from both of these mouse models and utilized them for 16S rRNA gene sequencing. We find that both diet-mediated and deletion-mediated BD result in the expansion of opportunistic microbes including *Klebsiella*, *Enterobacter*, and *Helicobacter*, at the expense of mucus-resident microbes including *Akkermansia*. Additionally, microbiome dysbiosis resulting from diet-mediated BD precedes the onset of the IBD-like phenotypic changes. Lastly, through the use of predictive metagenomics, we report that the resulting BD-linked microbiome perturbations exhibit increased biotin biosynthesis in addition to several other perturbed metabolic pathways. Altogether, these results demonstrate that biotin deficiency results in a specific microbiome composition, which may favor microbes capable of biotin synthesis and which may contribute to intestinal inflammation.

## 1. Introduction

Biotin is a water-soluble vitamin that plays an important role in human health and disease as a coenzyme for carboxylases used in several metabolic pathways as well as the cellular stress response, gene regulation, and immune responses [1,2,3,4,5]. Furthermore, biotin deficiency has been associated with diseases like Inflammatory Bowel Disease (IBD), Alcoholism, and increased gut epithelial permeability [6,7]. Intestinal absorption of biotin occurs via a carrier-mediated process that involves the sodium-dependent multivitamin transporter (SMVT) and the vitamin is provided from both dietary and gut microbiota sources [8]. The causal relationship between changes in the microbiome, vitamin status and colitis remains incompletely understood. For more than 30 years, our group has characterized different aspects of the SMVT, biotin-transporter system including its physiology, cell biology and regulation [9,10,11,12]. In earlier studies to explore how biotin levels impact the microbiome and colitis, we generated mice with an intestine-specific conditional knock-out of the biotin transporter (SMVT; Slc5a6) [13]. This model (SMVT-cKO) helped to confirm that SMVT is the only biotin transport system in the intestine, but the mice died within the first 7 weeks of life making it challenging to use this model for longer-term studies. Next, we developed an intestine-specific, inducible SMVT knockout mouse (SMVT-icKO) which allowed us to study the biotin transporter in adult mice and the consequence of its deletion [14]. We found that mice with such deletion to have increased fecal calprotectin, IL-1β, TNF-ɑ, IFN-ɣ and activation of the NLRP3 inflammasome. We complemented these studies with a model for diet-induced biotin deficiency where mice developed a similar phenotype [15]. In all models of biotin deficiency, histologic samples from the colon had features of IBD. Furthermore, we used the dietary-deficiency model and the dextran sulfate sodium model for colitis to show that biotin supplementation is protective and ameliorates murine colitis by preventing NF-κB activation. Finally, in the SMVT-icKO model, we used broad-spectrum antibiotics to normalize intestinal inflammation, proinflammatory cytokines, and altered mucosal integrity. This led us to hypothesize that deficiency in biotin may be inducing colitis by altering the gut microbiome.

Others have also noted phenotypic changes related to biotin-deficiency induced dysbiosis. For example, germ-free mice receiving a diet deficient in dietary biotin developed alopecia, but this effect was not seen in conventional mice on the same diet [16]. That study was published in 1997 when the importance of the microbiome was not fully appreciated and Hayashi et al. improved on the model in 2017 [17]. They treated mice with vancomycin to induce intestinal overgrowth of *Lactobacillus murinus*, a strain which lacks biotin biosynthesis genes and likely consumes biotin for its own growth. When these mice were put on a biotin deficient diet, they developed alopecia which could be reversed with biotin therapy. These findings create a direct link between the microbiome and pathology associated with biotin deficiency.

The current study further characterizes dysbiotic changes associated with biotin deficiency and colitis using two models for biotin deficiency. The dietary deficiency model eliminates biotin from the GI lumen using a biotin deficient diet that contains egg-whites. Egg-whites contain high levels of avidin that binds to biotin and prevents its absorption. In this model, localized biotin deficiency occurs before systemic deficiency and induces changes to the microbiome before phenotypic signs like hair loss and weight loss were noted. The second model used SMVT-icKO mice which develop systemic deficiency acutely while biotin is still available in the lumen. The microbiome changes in this model more closely represent communication between the host inflammatory response and the microbiome. We found that in the dietary model, bacterial genera including *Klebsiella* and *Lactobacillus* were more abundant and strongly associated with an IBD-like phenotype. In the SMVT-icKO mouse, enriched genera included *Helicobacter*, which can be an opportunistic pathogen. These results confirm that regardless of the source of biotin deficiency, it is strongly associated with dysbiosis and an IBD-like phenotype.

## 2. Materials and Methods

### 2.1. Dietary Biotin Deficiency Mouse Model

The mouse model for diet induced biotin deficiency has been previously described [15]. Briefly, C57BL/6J mice purchased from Jackson Laboratories (Ellsworth, ME, USA) were housed at University of California Irvine in compliance with all University Laboratory Animal Guidelines. The biotin deficiency group received a diet with no added biotin and the protein source was 300 g/kg spray-dried, egg-white solids (TD.81079, Envigo, Indianapolis, IN, USA). The control group had the same diet, but supplemented with 0.004 g/kg biotin added to the feed (TD.97126, Envigo) or 1 mM biotin supplemented to the water supply. Mice received the diet *ad libitum* and were on these diets for up to 12 weeks.

#### 2.1.1. Inducible Biotin Deficiency Mouse Model

The SMVT-icKO mouse model has been previously described [14]. Briefly, *CreERT2+/SMVT-LoxP+/+* and their littermate controls *CreERT2+/SMVT-LoxP+/−* were housed at University of California Irvine in compliance with all University Laboratory Animal Guidelines. To induce deletion of SMVT, mice received tamoxifen (2 mg/mouse) dissolved in corn oil intraperitoneally on days 0, 1 and 2.

#### 2.1.2. Histologic Analysis

H&E-stained sections from the colon of mice were scored based on a modification to previously published methods [15]. Briefly, the score is the sum of: severity and extent of inflammatory cell infiltrate (0–3), epithelial changes including hyperplasia and goblet-cell loss (0–3), and changes to mucosal architecture including erosion and ulceration (0–3), with a maximum score of 9.

#### 2.1.3. Fecal Calprotectin Measurement

Fecal calprotectin was quantified by ELISA using the DuoSet Mouse S100A8/S100A9 Heterodimer kit (R&D Systems, Minneapolis, MN, USA) according to the manufacturer’s recommendations. Briefly, a stool was collected, weighed, and immediately frozen. At the time of assay, the stool is solubilized in fecal extraction buffer (0.1 mol/L Tris, 0.15 mol/L MaCl, 1.0 mol/L urea, 10 mmol/L CaCl_2_, 0.1 mol/L citric acid, 5 g/L bovine serum albumin, 0.25 mmol/L thimerosal, pH 8; Hycult Biotech, Wayne, PA, USA). The dilution with the extraction buffer was calculated assuming a density of 1 mg/mL.

### 2.2. Microbiome Analysis

#### 2.2.1. 16S rRNA Gene Sequencing and Library Preparation

Stool, colonic mucosal, and small intestinal luminal and mucosal samples of mice on biotin-deficient or control diets were processed by the UCLA Microbiome Core. DNA was extracted from each sample with the Zymobiomics DNA Miniprep Kit (cat#D4300) according to the vendor’s provided protocol. The extracted DNA underwent amplification of the V4 hypervariable region of the 16S ribosomal RNA gene using 515F and 806R barcoded primers as previously described [18]. Amplified products were purified using the ZR-96 DNA Clean & Concentrator-5 (cat#D4024). Samples were pooled with normalized quantities of DNA. Gel extraction, using 1.5% agarose gel and the Thermo Scientific GeneJET Gel Extraction Kit (cat#K0691), was utilized to purify the DNA library by excising the approximately 380 base pair fragment. 2 × 250 bp sequencing was performed at the UCLA Technology Center for Genomics & Bioinformatics on Illumina MiSeq using a v2 reagent kit, with a 25% PhiX spike-in.

Stool and colonic tissue samples flushed with phosphate-buffered saline (PBS) of mice on biotin-deficient or control diets in the longitudinal experiment were sent to Zymo Research for library preparation and sequencing. Amplification of the V3-V4 region of the 16S rRNA gene was performed with Zymo’s custom designed primer set, the Quick-16S™ Primer Set V3-V4 (Zymo Research, Irvine, CA, USA). Amplicon libraries were sequenced on Illumina MiSeq with a v3 reagent kit. The sequencing was performed with 10% PhiX spike-in.

Stool and colonic tissue samples flushed with PBS from SMVT-icKO mice were processed by the UCI Microbiome Initiative. Briefly, DNA was extracted with the Zymobiomics DNA Miniprep Kit and amplified at the V4-V5 hypervariable region of the 16S rRNA gene. PCR was performed using the EMP primers (515F (barcoded) and 926R) [19,20]. Sequencing was performed by UC Irvine GHTF using a MiSeq v3 chemistry with a PE300 sequencing length.

#### 2.2.2. Sequence Data Pre-Processing

Quality control, merging of forward and reverse reads, and denoising was performed utilizing the DADA2 pipeline, resulting in an amplicon sequence variant (ASV) x samples count table [21]. Taxonomy assignment for the dietary biotin deficiency and supplementation experiment was performed by Zymo utilizing Zymo’s classifier trained on an internally curated database. Taxonomy assignment for the dietary biotin deficiency and SMVT-icKO models was performed utilizing the naive Bayesian classifier implemented in the assignTaxonomy function of DADA2 using the Silva v132 database [22].

Using QIIME 2- 2019.10, the ASV table was split into datasets of different sample types. These datasets were rarefied to adjust for uneven sequencing depth prior to calculating shannon and observed-otus (which is equivalent to the total number of unique ASVs) indices for alpha diversity. The unrarefied ASV tables were additionally prevalence filtered to only the sequences present in at least three mice, prior to generating robust Aitchison distance matrices with the QIIME DEICODE plugin (https://library.qiime2.org/plugins/deicode/19/, accessed on 19 October 2022) and performing principal component analysis. Lastly, the unrarefied ASV tables were collapsed into higher-order phylogeny. All QIIME artifacts were subsequently exported to .tsv files for further analysis in R. QIIME commands utilized were wrapped in our custom bash scripts found on https://github.com/julianneyang/fast-16s-analysis, accessed on 19 October 2022.

#### 2.2.3. Data Analysis, Statistics, and Figure Generation

All data analysis was performed in the RStudio IDE (version 1.3.959) running R version 4.0.2. Packages critical to this work were *EnhancedVolcano*, *ggplot2*, *ggpubr*, *cowplot*, *viridis*, and *rlang* for figure generation, and packages *nlme*, *Maaslin2*, and *vegan* were utilized for analysis. *T*-tests and non-parametric Wilcoxon rank-sum tests were performed to compare distributions of alpha-diversity indices between groups. Adonis PERMANOVA was utilized to assess whether there were significant differences in beta-diversity as assessed through robust Aitchison distances between groups, while a repeat-measures aware PERMANOVA written by Lloyd-Price et al. was used to assess differences in microbiome beta-diversity in the stool longitudinal datasets [23]. Differential abundance testing was performed on a per-genus basis through fitting the log-transformed, total sum scaling-normalized counts to linear models implemented in MaAslin2 with Diet (encoding BD vs. CD) or Genotype (encoding SMVT-icKO or WT) as the primary predictor variable and adjusting for relevant covariates. Linear models also incorporated MouseID as a random effect in the stool longitudinal datasets. Genera were determined as significantly associated with Diet or Genotype if the FDR-corrected *p*-value (q-value) met the significance threshold of <0.05. For all inferential statistics, the alpha was set to 0.05.

For predictive metagenomics, the 16S rRNA sequencing data was utilized as inputs for the PICRUST2 algorithm, which predicts functions of a bacterial community based on 16S marker sequences [24]. To evaluate whether the predicted MetaCyc pathway outputs were significantly associated with Diet or Genotype, we applied the same linear models for genus association testing, as described above. Pathway annotations were applied using the add_descriptions.py script provided on the PICRUST2 repository maintained by developer Gavin Douglas, https://github.com/picrust/picrust2/wiki, accessed on 1 November 2022.

Statistics, R scripts, and commands run from the terminal accompanying this study can be found on the following public GitHub repository, https://github.com/julianneyang/biotindeficiency, accessed on 1 November 2022. The graphical abstract was created with BioRender on BioRender.com.

## 3. Results

### 3.1. Dietary Biotin-Deficiency Is Associated with a Distinct Microbiome Composition along the Gastrointestinal Tract

**Figure 1:**
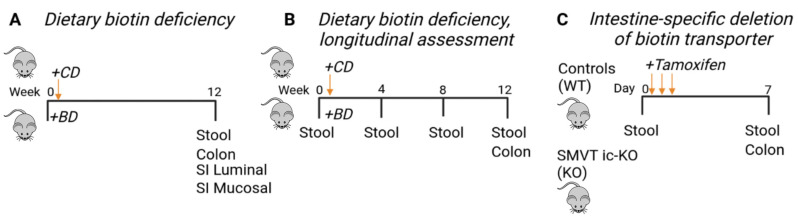
Study design and schematic illustrating the samples collected from the three experiments which were utilized for 16S rRNA gene sequencing. (**A**) Mice were placed on either a control diet (CD, *n* = 10) or biotin-deficient diet (BD, *n* = 7). At euthanasia, luminal and mucosal-adherent samples from the small intestine (SI), colon samples, and stool samples were collected for 16S rRNA amplicon sequencing. (**B**) Mice were placed on a CD (*n* = 8) or BD (*n* = 4). Baseline stool collected prior to the diet treatment, stool samples collected at weeks 4, 8, as well as stool and colon samples collected at Week 12, were utilized for microbiomics. (**C**) Baseline stool was collected from SMVT-icKO (KO, *n* = 4) or their littermate controls (WT, *n* = 4). Following tamoxifen treatment on Days 0, 1, and 2 to induce deletion of SMVT, stool and colonic samples were collected at Day 7 and utilized for microbiomics.

We previously reported that dietary biotin deficiency results in an IBD-like phenotype, including weight loss, bloody stool, and histologic changes including severe lymphocyte infiltration, loss of goblet cells and ulceration in the colon and small intestine [15]. To elaborate on the evidence supporting BD-induced microbiome dysbiosis as a contributor to colitis, we studied two models for inducing biotin deficiency. In the first model, we placed mice on a biotin-deficient (BD) or a control diet (CD) for 12 weeks to determine if microbiome dysbiosis accompanies IBD-like phenotypes (Figure 1A) and the kinetics at which this occurs (Figure 1B). Similarly, we studied the effects of deletion of the biotin transporter which leads to severe deficiency within a week (Figure 1C). In all models, a combination of luminal and mucosal samples from the small intestine and/or colon, as well as stool samples were harvested from the mice and utilized for 16S rRNA amplicon sequencing.

**Figure 2:**
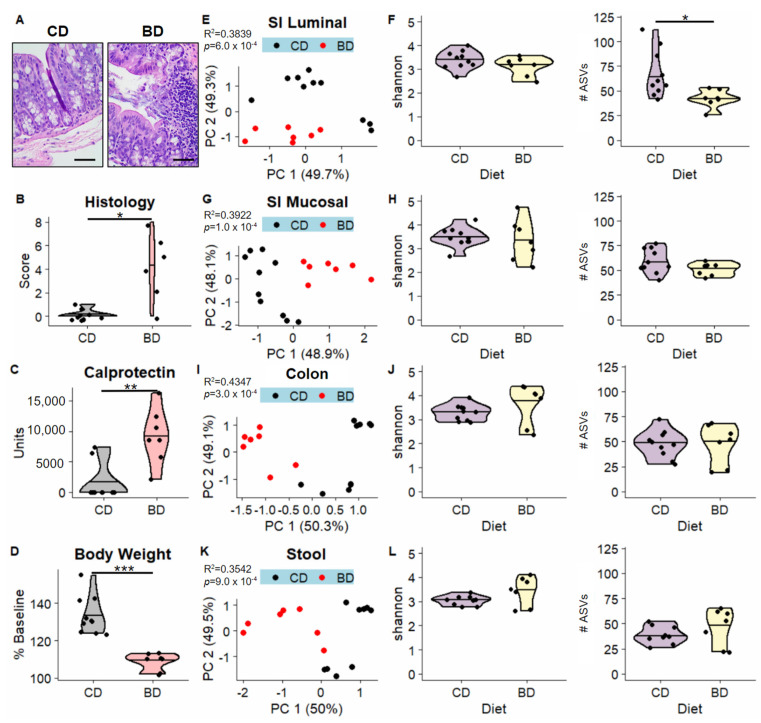
Dietary deficiency results in an IBD-like phenotype and perturbations in microbiome diversity. (**A**) Representative hematoxylin and eosin -stained colon images from control diet (CD) and biotin-deficient diet (BD) mice. The scale bar represents 50 microns. (**B**–**D**) Comparison of colonic histology scores, fecal calprotectin levels measured in units of pg calprotectin/mg stool, and percent baseline body weight between CD and BD groups. Beta-diversity was visualized using principal coordinates analysis for the small intestinal (SI) luminal (**E**), SI mucosal-adherent (**G**), colon (**I**), and stool (**K**) samples, with one dot representing one sample and colored according to Diet (CD or BD). R^2^ and *p* values associated with Diet are labeled in the top left corner of each plot. Violin plots compare two alpha-diversity metrics, shannon (left) and #ASVs (right) between diet groups, separated into SI luminal (**F**), SI mucosal (**H**), colon (**J**), and stool (**L**) samples. Significance was assessed through Student’s *t*-test, * *p* < 0.05, ** *p* < 0.01, *** *p* < 0.001.

Consistent with our previous findings, BD mice developed severe colonic inflammation when compared to CD mice. BD mice develop histologic changes characterized by mucosal and submucosal lymphocyte infiltration, hyperplasia, loss of goblet cells and erosion/ulceration (Figure 2A). These mice have significant changes in histology scores and increased levels of the inflammatory biomarker, calprotectin (Figure 2B,C). Additionally, BD mice have reduced percent body weight changes compared to CD mice (Figure 2D).

We discovered that, across all sampled sites of the GI tract, diet significantly accounted for 35 to 43% of the variation in microbiome beta-diversity as assessed through robust Aitchison distances (Figure 2E,G,I,K). There were no differences in species richness and evenness as assessed by the Shannon index, though we note a significant reduction in the total number of ASVs in the BD small intestinal luminal samples compared to the CD small intestinal luminal samples (Figure 2F,H,J,L).

**Figure 3:**
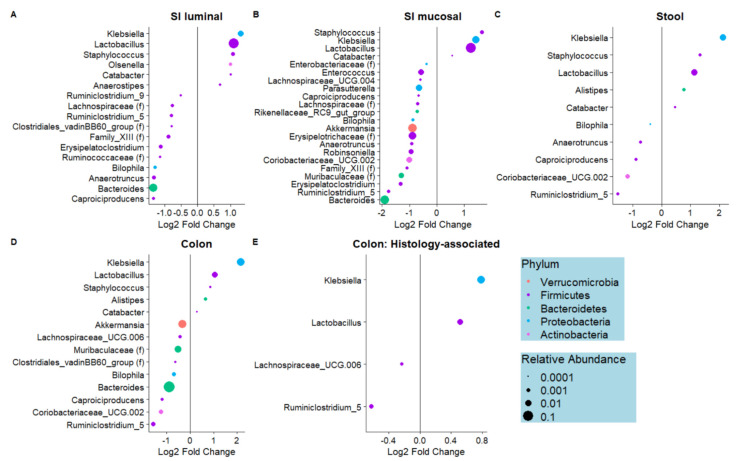
Significant dietary biotin-deficiency-associated and histology-associated genera. Per-genus association testing was performed utilizing linear models fitted to log-transformed, TSS normalized count data with Sex and Diet as covariates (**A**–**D**), or Sex and Histology Score as covariates (**E**). Modeling was performed separately for samples obtained from each region of the GI tract; small intestinal (SI) luminal (**A**), SI mucosal-adherent (**B**), stool (**C**), and colon (**D**,**E**). Genera which were significantly differentially abundant (*q* < 0.05) in the biotin-deficient (BD) group compared to control diet (CD) are represented as dots colored by phylum and sized according to relative abundance, with the legend for all plots shown in (**E**). Relative to CD, genera with positive log2foldchange are enriched in BD, whereas genera with a negative log2foldchange are depleted in BD.

Several bacteria genera were differentially abundant in the BD mice compared to the CD mice; in all sampled sites, *Klebsiella*, *Lactobacillus*, *Staphylococcus*, and *Catabacter* were enriched in BD relative to CD mice (Figure 3A–D). Notably, *Klebsiella* and *Lactobacillus* were also positively associated with histology score in the cecum samples, suggesting increased inflammation in the presence of these genera, while *Lachnospiraceae UCG-006* and *Ruminiclostridium_5* abundance was negatively associated with histology (Figure 3E). No other genera were associated with histology in any of the other datasets, with the exception of *Catabacter* in the small intestinal luminal samples, which was positively associated with histology (log2(BD/CD) = 0.504, *q* = 2.84 × 10^3^). In the small intestinal mucosal and colonic datasets, known mucin-degrading microbes *Bacteroides* and *Akkermansia* were depleted in the BD mice compared to the CD mice. Taken together, these results demonstrate, for the first time, that dietary biotin deficiency results in a distinct microbiome composition along multiple sites of the GI tract, and several of these microbes are positively associated with inflammation as assessed through histological scoring.

### 3.2. Dietary Biotin-Deficiency Effects on the Microbiome Precede IBD-Linked Body Weight Loss and Colonic Inflammation

**Figure 4:**
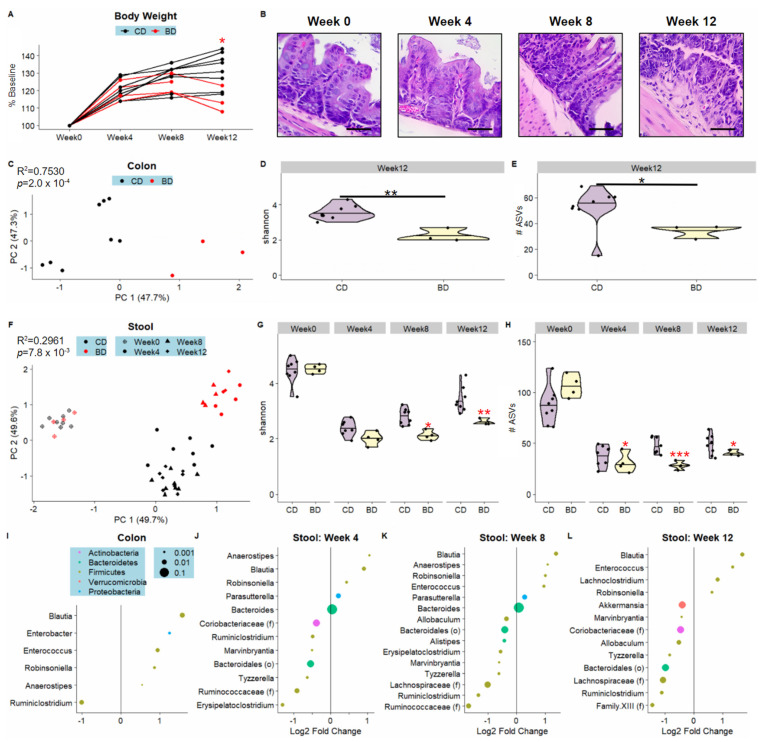
Microbiome dysbiosis induced by dietary biotin deficiency occurs prior to changes in body weight and intestinal pathology. (**A**) Line graph showing changes in body weight at the study timepoints, with each line representing one mouse. (**B**) Representative hematoxylin and eosin-stained colon images of biotin-deficient diet (BD) mice at week 0, 4, 8 and 12. The scale bar represents 50 microns. Beta-diversity was visualized using principal coordinates analysis in the colon (**C**) and stool (**F**), with one dot representing one sample and colored according to Diet (CD, BD). R^2^ and *p*-values associated with Diet are labeled in the upper left corner of each plot. Violin plots compare alpha-diversity metrics, shannon (left) and #ASVs (right) between diet groups in the colon (**D**,**E**) and stool (**G**,**H**). For the colon data, significance was assessed through Student’s *t*-test, * *p* < 0.05, ** *p* < 0.01. For the longitudinal data, significance was assessed through linear mixed-effects models (LMEM) with Diet as a fixed effect and MouseID as a random effect. A red asterisk indicates that the BD trajectory is significantly different from the CD trajectory; * *p* < 0.05, ** *p* < 0.01, *** *p* < 0.001. Per-genus association testing for the colon (**I**), stool week 4 (**J**), stool week 8 (**K**), and stool week 12 (**L**) was performed using linear models fitted to log-transformed, TSS normalized count data with Diet as the predictor variable. Genera which were significantly differentially abundant (*q* < 0.05) in BD compared to CD are represented as dots colored by Diet and sized according to relative abundance, with the legend for all differential taxon plots shown in (**I**). Relative to the control group, genera with positive log2foldchange are enriched in BD.

Accumulating evidence in colitis mouse models and humanized mice implicate microbiota as key drivers of inflammation [25,26,27]. We thus hypothesized that the microbiome changes would precede inflammation onset and loss in body weight characteristic of this model. We followed mice placed on either a CD or BD over the course of 12 weeks (Figure 1B). We found that the trajectory of body weight differed significantly between CD and BD only at week 12 (Figure 4A). Consistent with this observation, in a parallel cohort of mice, we found that histologic changes were only significant at week 12, but not at intermediate timepoints week 4 or 8 (Figure 4B). To investigate how the microbiome changes over the duration of the experiment, we utilized stool samples collected from Weeks 0, 4, 8, and 12 and analyzed them longitudinally, in addition to comparing the Week 12 colonic samples between diet groups (Figure 1B). In the colonic samples, diet had a large and significant effect, explaining 75% of the variation in microbiome beta-diversity (Figure 4C). Additionally, the BD colonic samples had significantly reduced species evenness and richness compared to CD as assessed through the Shannon index and total number of ASVs (Figure 4D,E). Among the genera enriched in the colons of BD relative to CD mice were *Blautia*, *Enterobacter*, and *Enterococcus,* while *Ruminiclostridium* was depleted (Figure 4I).

In the longitudinal assessment of stool samples, robust Aitchison principal coordinates analysis (PCoA) revealed distinct BD and CD clusters at weeks 4, 8, and 12 (Figure 4F). Samples collected at baseline week 0 all clustered together, which reflects the collection of stool prior to the start of the experimental diets. In terms of species richness and evenness (Shannon), BD stool samples exhibited a significantly reduced positive trajectory over the time course of the experiment compared to CD at Weeks 8 and 12. (Figure 4G). Considering only the total number of ASVs, BD stool samples had reduced trajectories at Weeks 4, 8, and 12 (Figure 4H). At Weeks 4 and 8—prior to the onset of intestinal inflammation—12 and 15 genera were found to be significantly differentially abundant, with 10 genera identified in both timepoints (Figure 4J,K). 13 genera were identified as significantly differentially abundant at week 12 (Figure 4L). In weeks 4 and 8, *Parasutterella* from phylum Proteobacteria was enriched in BD compared to CD (Figure 4K). *Blautia* was significantly enriched in BD at all timepoints (Figure 4J,L). At week 12, there was reduced abundance of *Akkermansia* in BD relative to CD (Figure 4L). These experiments illustrate that the onset of microbiome dysbiosis predates the onset of colitis-linked changes such as body weight loss and intestinal pathology.

### 3.3. Intestine-Specific Deletion of Biotin Transporter Results in Perturbations to the Colonic and Fecal Microbiome

**Figure 5:**
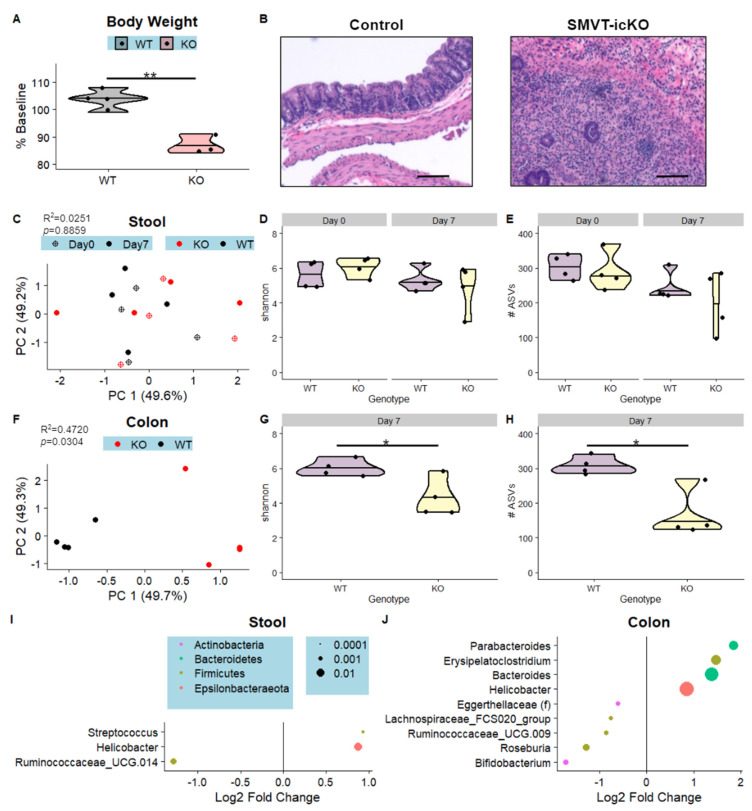
Host biotin deficiency induced by intestine-specific biotin transporter deletion (SMVT-icKO) results in intestinal pathology and microbiome composition consistent with inflammatory changes. (**A**) Violin plot comparing % baseline body weight between genotypes (SMVT-icKO—KO, littermate controls—WT). (**B**) Representative hematoxylin and eosin-stained colon images from WT and KO mice. The scale bar represents 100 microns. Beta-diversity visualized through principal coordinates analysis plots is shown for stool (**C**) and colon (**F**) samples. R^2^ and *p*-values associated with genotype were determined by PERMANOVA (colon subset) or PERMANOVA accounting for repeated measures (stool subset) and are displayed in the upper left corners of each plot. Violin plots compare two alpha-diversity metrics, shannon and (right) between genotypes for stool (**D**,**E**) and colon (**G**,**H**). Significance was assessed through Student’s *t*-test, * *p* < 0.05 ** *p* < 0.01. Per-genus association testing was performed utilizing linear mixed-effect models fitted to log-transformed, TSS normalized count data with Timepoint and Diet as fixed effects and MouseID as a random effect for the stool subset (**I**), or a linear model fitted to log-transformed, TSS normalized count data with Genotype as the variable for the Colon subset (**J**). Genera which were significantly differentially abundant in the KO group compared to WT (*q* < 0.05) are represented as dots colored by Diet and sized according to relative abundance, with the legend for both plots shown in (**I**). Relative to the control group, genera with positive log2foldchange are enriched in KO relative to WT.

To determine the contribution of host biotin deficiency on the microbiome, we utilized the SMVT-icKO line, which models biotin deficiency through deletion of the SMVT transporter in the intestinal epithelium. As described above, this mouse develops severe disease shortly after the SMVT biotin transporter is conditionally deleted from the intestine via tamoxifen induction [14]. By day 7, following deletion, these mice develop weight loss, rectal bleeding and intestinal inflammation. Four pairs of SMVT-icKO mice (KO) and their cage-matched littermate controls (WT) received three doses of tamoxifen on days 0, 1, and 2, and were tracked for 7 days (Figure 1C). At day 7, consistent with our previous reports on this model, KO mice had significantly reduced body weight and widespread mucosal, submucosal, and transmural colonic inflammation compared to WT mice (Figure 5A,B). Following euthanasia, stool and colon samples were utilized for 16S rRNA gene sequencing, and the microbiome profiles were compared between KO and WT mice (Figure 1C).

Microbiomics of the stool samples revealed lack of KO-linked changes in microbiome beta- or alpha- diversity after accounting for differences at baseline, though genera *Streptococcus* and *Helicobacter* were enriched in KO mice while *Ruminococcaceae_UCG-014* was depleted in KO relative to WT mice (Figure 5C–E,I). The colonic samples exhibited more KO-linked perturbations in the microbiome, with genotype significantly accounting for 47% of the variation in beta-diversity, KO-linked reductions in both species richness (number of ASVs) and evenness (Shannon), and 9 genera which were significantly differentially abundant in the KO group compared to the WT group (Figure 5F,G,H,J). *Bacteroides* and *Helicobacter* were among the genera enriched in the KO mice, while *Roseburia* and *Bifidobacterium* were among the genera depleted in KO mice relative to controls (Figure 5J). Thus, these results demonstrate that deficits in host biotin absorption perturb the gastrointestinal microbiome, and the dysbiosis is more clearly detected in the colon than in the stool.

### 3.4. Microbes Associated with Biotin Deficiency Are Predicted to Exhibit Increased Biotin Biosynthetic Capabilities

We then utilized the 16S rRNA sequencing data from both the dietary BD and inhibited host absorption BD models as inputs to the PICRUST2 metagenome prediction algorithm [24]. To identify functional MetaCyc pathways that were significantly differentially abundant in BD vs. non-BD mice, we utilized the same model structures as were used for per-genus association testing.

**Figure 6:**
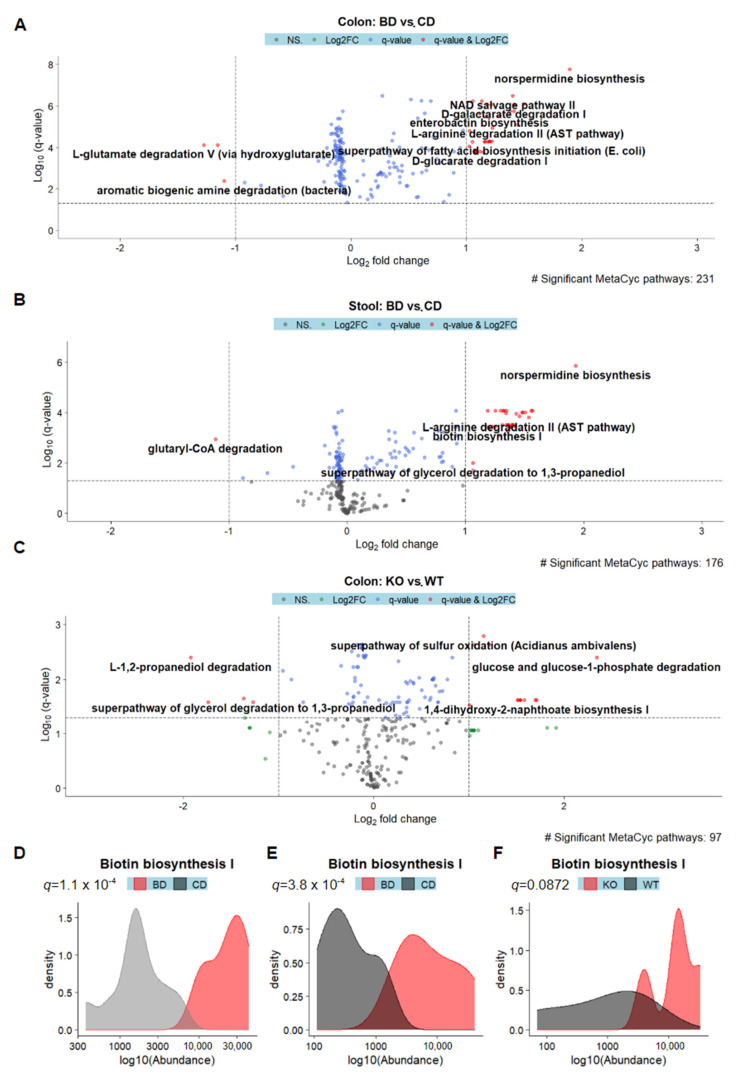
Predictive metagenomics of the dietary biotin deficiency and host biotin deficiency models reveal perturbed functional pathways and biotin biosynthesis. Volcano plots illustrating significantly differentially abundant Metacyc pathways which are associated with dietary biotin-deficiency (BD) in the colon (**A**) and stool (**B**) datasets or host BD in colon (**C**), with each dot representing a single pathway. Labels are not shown for all pathways. Density plots showing the log10-transformed predicted abundances of the Biotin biosynthesis I pathway in the colon (**D**) and stool (**E**) of dietary BD mice, or of the colon in host BD mice (**F**).

In the dietary BD model, 231 and 176 MetaCyc pathways were identified as being significantly differentially abundant in the colon and stool datasets, respectively (Figure 6A,B). Pathways which were enriched in the colons of dietary BD mice included norspermidine biosynthesis, enterobactin biosynthesis, and superpathway of fatty acid biosynthesis initiation, while pathways that were depleted included L-glutamate degradation V and aromatic biogenic amine degradation (Figure 6A). In the stool dataset, norspermidine biosynthesis was among the enriched, and glutaryl-CoA degradation was among the depleted (Figure 6B). Biotin biosynthesis was significantly enriched in both the colon and stool datasets (Figure 6D,E).

In the host BD model, 97 MetaCyc pathways were differentially enriched in the colon, but no pathways were identified as being differentially enriched in the stool, consistent with the lack of global changes in microbiome diversity (Figure 6C). Among the pathways that were enriched were glucose and glucose-1-phosphate degradation, while L-1,2-propanediol degradation was depleted in the colons of KO mice compared to controls (Figure 6C). Biotin biosynthesis was increased in KO relative to WT mice, though this difference was not statistically significant after correction for multiple hypothesis testing (*q* = 0.0872). These results suggest that biotin deficiency perturbs the normal functions of intestinal microbiome, perhaps by selecting for microbes with biotin synthesis capabilities.

## 4. Discussion

This report is the first in-depth analysis of microbiome changes associated with BD-induced colitis. We first demonstrate that dietary BD results in gut microbiome dysbiosis, which is associated with an IBD-like phenotype. We observed along several sites of the GI tract that genus *Klebsiella* was enriched in BD mice relative to CD mice in addition to being positively correlated with intestinal inflammation. This is consistent with other reports showing intestinal colonization of germ-free mice with oral *Klebsiella* led to induction of gut Th1 cell populations and inflammation [28]. In the TNBS colitis mouse model, oral administration of *K. pneumoniae* increased pro-inflammatory cytokine expression and lipid peroxidation in the colon while reducing the expression of tight-junction proteins [29]. Another gut-atypical microbe which had increased abundance in BD compared to CD mice was *Staphylococcus,* which may support existing observations of increased intestinal expansion of oral bacteria in colitis patients [30]. We also note that the abundances of the mucosa-resident microbe *Ruminiclostridium_5* in addition to mucin degraders *Bacteroides* and *Akkermansia* and *Lachnospiraceae_UCG-006* were reduced in BD compared to CD mice, which may be related to the thinning of the mucus layer [31,32]. Reduction of core mucins occurs early on in patients with ulcerative colitis [33]. In colon explant cultures exposed to dextran sulfate sodium, increased permeabilization and bacterial infiltration were observed prior to the onset of inflammation, illustrating a link between mucus layer thinning and inflammation [34]. Consistent with these observations, we identified *Ruminiclostridium_5* and *Lachnospiraceae_UCG-006* as microbes negatively correlated with inflammation as assessed through histological scoring.

Microbes depend on biotin for growth and survival, and either synthesize biotin or scavenge it from the environment. Biotin-dependent enzymes, such as acyl-coA carboxylase and pyruvate carboxylase, are involved in critical metabolic pathways such as fatty acid synthesis and synthesis of the tricarboxylic acid cycle intermediate oxaloacetate [35]. Biotin synthesis in microbes occurs through synthesis of a pimelate precursor followed by the assembly of the biotin rings [36]. Based on analysis of the annotated genomes of 256 human gut microbes, the majority of Bacteroidetes and Proteobacteria are capable of biotin synthesis, while only 5 genomes belonging to class Clostridia and *B. subtilis* from phylum Firmicutes possess biotin synthesis genes [37]. While biotin synthesis genes were completely absent from Actinobacteria, members of this phylum possessed a biotin transporter, suggesting a need for biotin and cooperation between microbes [37]. Though we identify both BD-enriched and depleted genera belonging to phylum Bacteroidetes, BD-enriched microbes *Klebsiella* and *Lactobacillus* are capable of biotin synthesis and biotin transport, respectively [38,39,40]. Additionally, taxonomy-agnostic predictive metagenomics revealed that biotin biosynthesis was enriched in BD relative to CD mice. Among the other pathways enriched in BD mice was the biotin-utilizing superpathway of fatty acid synthesis initiation, while other pathways that were enriched suggested microbial adaptation to oxidative stress, such as enterobactin (a siderophore) biosynthesis and norspermidine biosynthesis (biofilm formation) [41,42].

We next utilized a longitudinal model of dietary biotin deficiency and identified that BD-linked microbiome dysbiosis occurred as early as 4 weeks after introduction of the diet, prior to the initiation of mucosal lymphocyte infiltration at 12 weeks and phenotypic changes like body weight loss. In this dataset, the genera *Enterobacter* and *Parasutterella* from phylum Proteobacteria were enriched in BD at weeks 4 and 8, but not 12. Aside from possessing biotin synthesis capabilities, the expansion of Proteobacteria is observed in IBD patients and in mouse models of colitis [43]. Interestingly, *Enterobacter* and *Parasutterella* were not identified as being significantly enriched in BD at week 12 concomitant with the onset of inflammation, while *Akkermansia* was identified as being depleted at week 12 but not at weeks 4 or 8. We propose that the lack of biotin availability results in an early composition which favors biotin synthesizers and effective scavengers, and that the week 12 composition may be affected more by the inflammation in the local environment. However, additional studies would be needed to test this hypothesis. We acknowledge that there is not a consensus BD-associated microbial signature between the two dietary BD experiments. This may reflect differences in preparation of sequencing libraries, and is a limitation of this study.

Finally, to better understand host-microbe interactions in the competition for biotin, we studied the colonic and fecal microbiomes of SMVT-icKO (KO) and their littermate controls (WT). Following tamoxifen induction, these mice exhibited severe body weight loss and widespread colonic inflammation by day 7. The genus *Helicobacter* was enriched in both colon and stool samples of KO mice relative to WT mice. This may reflect both its characterization as biotin synthesizers and as a group containing several disease-associated opportunistic pathogens [37,44,45,46]. We identified an effect of genotype on microbiome composition as assessed through beta- and alpha-diversity in the colon samples but not the stool samples, suggesting that the effects of host biotin deficiency affect primarily the bacteria at the mucosal-luminal interface rather than the luminal bacteria. Supporting this notion, in the colon samples, there were 97 significantly differentially abundant predicted MetaCyc pathways while there were 0 such pathways in the stool dataset. This is in stark contrast to the dietary biotin deficiency model, where 231 and 176 differentially abundant pathways between diet groups were identified in the colon and stool datasets, respectively. Predicted biotin biosynthesis was enriched in KO relative to WT mice and was nominally significant, though the q-value did not fall below the alpha of 0.05 following Benjamini-Hochberg correction (*q* = 0.0872). Interestingly, predicted L1,2-propanediol degradation was reduced in KO relative to WT mice. Under fermentation conditions, propionate is one of the products of this pathway. Evidence supports an anti-inflammatory role for propionate in the intestines, with a recent study demonstrating that it can suppress IL-17 production by gamma-delta T cells [47]. Taken together, these results suggest that biotin deficiency mediated through inhibition of host biotin absorption provides an environment well-suited to biotin-synthesizing microbes at the luminal-mucosal interface at the expense of other commensals, which may contribute to the severe intestinal inflammation observed in these mice.

In summary, we illustrate clear effects of both diet-induced BD and impaired absorption-mediated BD on the gut microbiome, and highlight the BD-linked enrichment of several opportunistic microbes across these datasets. We also demonstrate that microbiome perturbations occur at least 8 weeks prior to the onset of IBD-linked phenology. Utilizing predictive metagenomics, we find that microbial biotin biosynthesis is more abundant in both diet-induced and absorption-mediated BD. These findings will facilitate future experiments elucidating the role of the BD-linked microbiota in inflammation and may lead to new therapeutics in the future.

## Data Availability

The raw 16S rRNA gene sequencing data are available in the NCBI Bioproject repository (https://www.ncbi.nlm.nih.gov/bioproject/PRJNA904819, accessed on 20 November 2022). Preprocessed sequencing data, metadata, scripts, and details sufficient to replicate data preprocessing and analysis are publicly accessible on https://github.com/julianneyang/biotindeficiency, accessed on 1 November 2022.
